# Proconiini Sharpshooters of Argentina, with Notes on Its Distribution, Host Plants, and Natural Enemies

**DOI:** 10.1673/031.012.11601

**Published:** 2012-10-09

**Authors:** Susana L. Paradell, Eduardo G. Virla, Guillermo A. Logarzo, Gimena Dellapé

**Affiliations:** ^1^ Universidad Nacional de La Plata. Facultad de Ciencias Naturales y Museo. División Entomología. Paseo del Bosque s/n (1900), La Plata, Buenos Aires, Argentina; ^2^ PROIMI-Biotechnology, Biological Control Division, CONICET, Av. Belgrano y Pje Caseros (4000), San Miguel de Tucumán, Tucumán, Argentina; ^3^ USDA-ARS South American Biological Control Laboratory, Bolivar 1559 (1686), Hurlingham, Buenos Aires, Argentina

**Keywords:** Auchenorrhyncha, Cicadellidae, Cicadellinae, biogeographic provinces, bionomics, parasitoids

## Abstract

The American tribe Proconiini (Hemiptera: Cicadellidae: Cicadellinae) is one of the largest groups of xylem-feeding insects and includes the majority of the known vectors of xylem-born phytopathogenic organisms. The significance of the pathogens that this group transmits gives them an important role as pests, mostly for citrus fruit, grapes, and almonds. Knowledge of these Hemiptera in Argentina is insufficient and fragmentary. Thus one of the aims of this paper is to summarize the available information of the Proconiini sharpshooters in Argentina. In addition, 14 species are mentioned for the first time in the country, and new distributional data are given for 18 species. Thirty-four new associations between sharpshooters and host plants are recorded. New records of egg parasitoids are given for *Dechacona missionum, Molomea consolida, M. lineiceps*, and *Tapajosa similis*.

## Introduction

The Proconiini tribe (Hemiptera: Cicadellidae: Cicadellinae) is characterized by posterior legs at rest with knees not attaining posterior proepimeral margins, male pygofer and plates both usually with numerous evenly dispersed microsetae and antennal ledges usually protuberant in dorsal aspect (Young 1968). The tribe includes 422 species in 58 genera (McKamey 2007; Wilson et al. 2009) and is restricted to the New World, with only *Homalodisca vitripennis* having an extraAmerican distribution, after recent invasion of many islands in the Pacific Ocean (Pilkington et al. 2005). The sharpshooters are one of the largest groups of xylem-feeding insects and include the majority of the known vectors of xylem-born phytopathogenic organisms (Rakitov and Dietrich 2001; Redak et al. 2004).

The bacterium *Xylella fastidiosa* Wells (Xanthomonadales: Xanthomonadaceae) is a growing threat in the Neotropical region. It has been found in Mexico, Costa Rica, Venezuela, Paraguay, Brazil, and Argentina, and a clear association between the xylem-feeding habit of sharpshooters and their ability to transmit the bacterium has been observed (Hopkins 1989; Redak et al. 2004). Most South American countries are under high occurrence risk of this dangerous disease (Dellapé et al. 2011).

*Xylella fastidiosa* is the causal agent of diverse diseases: “Phony Peach Disease” (PPD), “Plum Leaf Scald” (PLS), “Pierce's Disease” (PD) of grapes, “Almond Leaf Scorch” (ALS), “Coffee Leaf Scorch” (CLS), and “Citrus Variegated Chlorosis” (CVC) (Gravena et al. 1998; Redak et al. 2004). The bacterium is a known threat in diverse regions of Argentina affecting almonds (ALS) in Catamarca and La Rioja provinces (Nome et al. 1992; Haelterman et al. 1996), as well as citrus orchards (CVC) in Misiones, Corrientes, and Entre Rios provinces (De Coll et al. 2000; Beltrán et al. 2004; Costa et al. 2009).

The information on faunistic aspects of Proconiini in Latin America is almost nonexistent, particularly in Argentina. In addition, most of the knowledge on proconiine vectors is derived from studies done in countries of the Nearctic region. Relatively few transmission studies have been carried out in the Neotropic, where the majority of sharpshooter species occur (Redak et al. 2004; Silva et al. 2007; Marucci et al. 2008).

In Argentina, the Proconiini tribe is mainly distributed in the northern region (Young 1968; Remes Lenicov et al. 1999; Virla et al. 2008), and there is almost no information regarding this economically important group. Only for few species is there available data, and most of them provide only distributional records and/or species association with commercial crops (Costilla et al. 1972; Remes Lenicov and Tesón 1985; Paradell 1995; Remes Lenicov et al. 1997, 1998, 1999, 2004; Virla et al. 2008).

To obtain a better understanding about this tribe in Argentina, this paper contributes new distributional records and/or host plants associations and parasitoids, and also summarizes the available data of the Proconiini sharpshooters in the country.

## Materials and Methods

Three sources were used to achieve the objectives: (1) bibliographical data; (2) specimens housed in the most important entomological collections of Argentina: Instituto Miguel Lillo (IMLA), Museo de Ciencias Naturales de La Plata (MLP), Museo Argentino de Ciencias Naturales “Bernardino Rivadavia” (MACN); and (3) research conducted by the working group.

**Figure 1.**
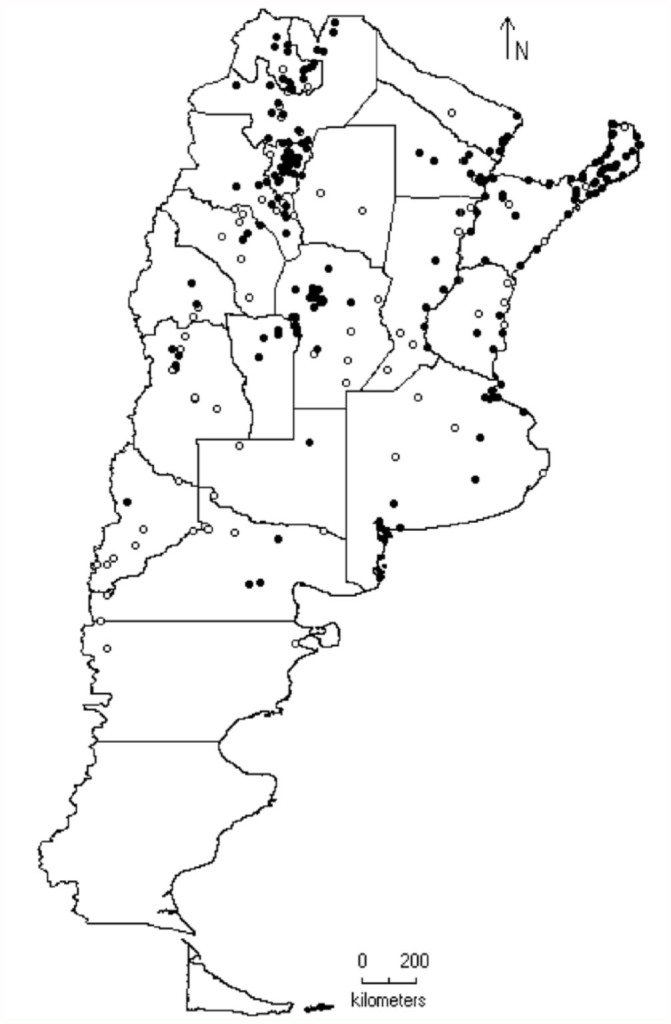
Distribution of the Proconiini sharpshooters in Argentina (black dots). White dots indicate sampled localities without occurrence of Proconiini species. High quality figures are available online.

112 sites in 21 provinces of Argentina were surveyed between 22° S and 44° S (Figure 1). Most of the sites were sampled by sweeping on diverse crops, its surrounding vegetation, and both anthropically-modified environments and pristine ones. In four occasions, Malaise traps and yellow pan traps were used as well (in Buenos Aires, Córdoba, and Rio Negro provinces). The specimens collected were preserved in 70% ethanol, and voucher specimens were deposited in the IMLA and MLP collections.

Both male and female genitalia of the species were prepared for microscopic examination using Young's techniques (1968). The parts were stored in microvials with glycerin. The specimens were identified using descriptions provided by Schröeder (1959), Young (1968), Emmrich (1975, 1984), Remes Lenicov et al. (1999), and Marucci et al. (2002). Data on *Anacuerna centrolinea* (Melichar) were obtained from the collection of the Staatliches Museum Für Naturkunde Stuttgart, Germany (SMNS).

An extensive distribution list of all species studied was made using both our own data, bibliographic records, and data of the specimens deposited in the Argentinean collections. Sharpshooter species were grouped into biogeographical regions proposed by Morrone (2001, 2006). The Jaccard Index was used to identify the similarities between the biogeographic provinces (Moreno 2001).

## Results

In the Argentinean territory, 40 species of Proconiini were found: 14 of them were reported for first time in Argentina, and 18 species had extended in geographic distribution. Also, new associations with host plants were found for six species of sharpshooters, and new records of parasitoid wasps for four species.

Below, 14 species of Proconiini recorded for the first time in Argentina are listed (Table 1, symbolized with an “A”):

***Acrogonia virescens*** (Metcalf). **Salta**: Abra Grande, Orán, 2♂♂ 1♀, III/67; 3♂♂ 1♀, 10/I28/II/67, Golbach Leg. (IMLA). **Misiones**: Eldorado, 1♂ 2♀♀, 31/X/2008, Logarzo and Palottini Legs. (MLP).

***Aulacizes basalts*** Walker. **Misiones**: San Antonio, 1♀, 7/XII/51, Willink and Monrós Legs.; Bernardo de Irigoyen, 1♀, 5/XII/51, Willink and Monrós Legs.; 2 de Mayo, 2♀♀, 30/XI/51, Willink and Monrós Legs.; Aristóbulo del Valle, 2♀♀, XI/51, Willink and Monrós Legs. (IMLA). **Corrientes**: Mburucuyá, 1♂, XI/57, BirabenLeg. (MLP).

***Aulacizes insistans*** (Walker). **Misiones**: Iguazú, 1♀, XII/57, Biraben Leg. (MLP); **Misiones**: 2♀♀, without other data (MACN).

***Aulacizes obsoleta*** Melichar. **Misiones**: Puerto Iguazú, 1♀, II/54, Willink and Golbach Legs. (IMLA); Iguazú, 2♀♀, XII/57, Biraben Leg.; Caraguatay, 1♂, I/60, Ronderos and Trotta Legs.; Eldorado (26° 25′ 40″ S, 54° 09′ 38.02″ W), 1♀, 30/X/2008, Logarzo and Palottini Legs. (MLP); 2♀♀, P. Aguirre Leg. (MACN).

***Cicciana latreittei*** (Distant). **Misiones**: Puerto Iguazú, 4♂♂, 20/XII/2001, Logarzo and Manrique Legs. (MLP).

***Diestostemma ptyloca*** Distant. **Misiones**: Iguazú, 3♂♂ 1♀, X/27; Iguazú, 1♂, X/77, Pepe Leg. (MACN).

***Oncometopia expansa*** Melichar. **Misiones**: 4♂♂ 1♀, III/1897, Venturi Leg.; Posadas, 1♂ (MACN); Eldorado, 2♂♂, XI/2008, Logarzo and Palottini Legs. (MLP).

***Oncometopia fusca*** Melichar. **Misiones**: Rep. Guaraní El Soberbio, 1♂, X/47, Viana Leg. (MACN); Loreto, 1♂, 21/IX/2003, Logarzo and Varone Legs. (MLP).

***Oncometopia rubescens*** Fowler. **Misiones**: Panambi, 2♂♂, X/51, Monrós and Willink Legs. (PMLA).

***Oncometopia venata*** Schröder. **Misiones**: Panambi, 1♂, 24/XI/51, Willink and Monrós Legs. (IMLA)

***Phera carbonaria*** (Melichar). **Misiones**: Iguazú, 3♂♂ 1♀, 10/XI/73, Tonsic and Willink Legs.; **Misiones**: 1♂, 4/IV/10, Jörgensen Leg.; 1♂, 31/VIII/10, Jörgensen Leg.; Parque Provincial Urugua-i, 1♂, 13/XII/57; San Javier, 1♂, 16/XII/57, Biraben Leg.; Iguazú, 1♂, XI/44, Biraben Leg. (MLP); **Misiones**: 3♂♂; Dep. Concepción-Sta. Maria, 1♂, X/46, Viana Leg. (MACN).

***Phera obtusifrons*** Fowler. **Misiones**: 2 de Mayo, 1♂, XI/73, Escobar and Claps Legs. (IMLA).

***Tretogonia callifera*** Melichar. **Formosa**: Clorinda, 7 specimens, XI/47; Mojón de Fierro, 2♂♂, XII/48, Golbach Leg. (IMLA).

***Tretogonia cribata*** Melichar. **Corrientes**: 9♂♂ 14♀♀, 2 without abdomen, II/59, Biraben Leg.; **Chaco**: 1♂ 2♀♀, III/59, Parko Leg. (MLP).

The geographic distributions of 18 species of Proconiini sharpshooters are extended as follows (Table 1, symbolized with “B” and “C”):

***Anacuerna centrolinea*** (Melichar). Jujuy: Morro de la Providencia, Quebrada de Humahuaca, Abra Pampa, Iturbe (IMLA). Salta: Cachipampa (SMNS).

***Aulacizes conspersa*** Walker. Misiones: Puerto Iguazú (IMLA), Caraguatay (MLP).

***Aulacizes quadripunctata*** (Germar). Misiones: San Pedro, Salto Encantado, San Antonio, Tobunas, Campo Grande, Caingua, Aristóbulo del Valle (IMLA); San Javier, 25 de Mayo (MACN); San Ignacio, 2 de Mayo, Eldorado (MLP).

***Dechacona missionum*** (Berg). Tucumán: Horco Molle, Monteras (MACN); La Higuera, Trancas. Salta: Pocitos, Urundel. Catamarca: Arroyo de Infanzón. Córdoba: Dique Los Molinos. Formosa: Estera La Florence, Clorinda (PMLA). Jujuy: Yuto, Gral. San Martín, Dique La Ciénaga. Salta: Bazán. Tucumán: Gonzalo. Misiones: Montecarlo. Corrientes: Empedrado (MLP).

***Egidemia speculifera*** (Walker). Misiones: Puerto Bemberg, San Pedro, 2 de Mayo (PMLA); Guaraní (MACN).

***Molomea consolida*** Schröder. Jujuy: Yuto, Aguas Calientes. Misiones: Montecarlo, Loreto, Garuhapé, Eldorado (MLP). Jujuy: Laguna de Yala, Aguas Calientes. Salta: Embarcación. Misiones: Puerto Bemberg, Iguazú, Oro Verde, San Javier, Panambi, 2 de Mayo. Tucumán. Catamarca: San Antonio. Salta: Tartagal, Aguaray (IMLA). Misiones: Obára, Posadas, Concepción, Santa María. Corrientes: Santo Tomé. Buenos Aires. Salta: Orán (MACN).

***Molomea lineiceps*** Young. Corrientes: Las Marías-Virasoro. Jujuy: Caimancito. Salta: Abra Grande. Tucumán: Las Talitas, El Bachi (PMLA). Buenos Aires: Isla Martin Garcia, Tigre. Corrientes: Monte Caseras, Santo Tomé. La Rioja. Santa Fé: Rosario (MACN). Tucumán: Horco Molle (MLP).

***Ochrostacta diadema*** (Burmeister). Corrientes: Manantiales, Sauce. Formosa: Misión Laishi, Mojón de Fierro (IMLA). Chaco: between Vedia and Pres. Roca, Bermejo River (MACN). Santa Fé: Guadalupe (MLP).

***Ochrostacta physocephala*** (Signoret). Misiones: San Ignacio, Pindapoy. Corrientes: Santo Tomé (MLP).

***Oncometopia facialis*** (Signoret). Misiones: San Javier, Iguazú, Arroyo Urugua-I, Santa Ana, San Antonio, Montecarlo, Aristóbulo del Valle, Panambi. Corrientes: Isla Iyupe Grande. Salta: El Morenillo, San Lorenzo. Tucumán: Cerro San Javier, Lules, Horco Molle, Chilcas, La Ramada. Catamarca: Aconquija, Concepción, Belén, El Rodeo (PMLA). Misiones: Concepción, Santa María. Jujuy: Quebrada Río Blanco (MACN). Misiones: Eldorado, Loreto (MLP).

***Oncometopia tucumana*** Schröder. Salta: Abra Grande, Aguaray, Tartagal, San Lorenzo. Catamarca: El Rodeo, Concepción, Belén. Misiones: Iguazú. Tucumán: San Javier, Cerro San Javier, Burruyacu, Chilcas (IMLA); Tucumán: Las Tipas (MLP).

***Pseudometopia amblardii*** (Signoret). Misiones: Iguazú (IMLA); Loreto (MLP).

***Tapajosa doeringi*** (Berg). Catamarca: El Suncho, Belén, El Alamito, El Rodeo. San Luis: San Francisco, San Martín, Merlo, Villa de Praga, Las Chacras, Cortaderas. Córdoba: Yacanto, Agua de Oro, La Cumbre, Punilla. Río Negro: Choele Choel (PMLA). Córdoba: Calamuchita, El Sauce, Argüello, San Javier. Buenos Aires: San Blas, Bahía Bianca. La Pampa: Conelho. Misiones. Formosa (MACN). Buenos Aires: Sierra de la Ventana, Monte Hermoso. Catamarca: Chumbicha (MLP).

***Tapajosa rubromarginata*** (Signoret). Jujuy: San Salvador, Gral. San Martín. Salta: Orán, Chalicán. Córdoba: Los Molinos, Huerta Grande. Entre Ríos: Concepción del Uruguay. Buenos Aires: Magdalena. Mendoza: Tunuyán (MLP). Jujuy: Calilegua. Salta. Chaco: Resistencia. Córdoba: Calamuchita, El Jag¨el, El Sauce, Arg¨ello, La Paz, La Falda, Alta Gracia. Santa Fé: Garay. Buenos Aires: Rosas FC Sud, Tandil. Corrientes: Monte Caseras. Mendoza: Cacheuta. Neuquén: Loncopué. Río Negro: Río Valcheta (MACN). Salta: Cafayate. Catamarca: Aconquija, El Rodeo. Tucumán: Monteras, Acheral, Aguadita, El Siambon, Monte Bello. San Juan: San Martín. San Luis: Cortaderas. Formosa: Misión Laishi, Clorinda. Misiones: Timbó, San Vicente, Puerto Bemberg. Corrientes: Paso de los Libres, Manantiales. Córdoba: Cabania, Agua de Oro, Dique Los Molinos. Santa Fé: La Gallareta, Villa Ana (IMLA).

***Tapajosa similis*** (Melichar). Jujuy: La Isla. Salta: Cafayate, Campo Quijano, Coronel Moldes. Catamarca: El Rodeo, Arroyo de Infanzón, El Alto. Tucumán: La Mezada, Horco Molle, Trancas, San Pedro de Colalao, Montebello, Río Chico, Tafí Viejo. Entre Ríos: Gualeguaychú (PMLA). Misiones. Salta. Chaco (MACN). Tucumán: Las Tipas (MLP).

***Teletusa limpida*** (Signoret). Misiones: Puerto Bemberg, Puerto Iguazú (IMLA).

***Tretogonia bergi*** Young. Misiones (MACN).

***Tretogonia notatifrons*** Melichar. Formosa: Clorinda, Misión Laishi, Mojón de Fierro. Chaco: Colonia Benítez. Misiones: Apóstoles, San José. Corrientes: Manantiales (IMLA). Chaco: Sáenz Peña, Resistencia, Barranqueras, Zapallar. Formosa: Las Ocas, El Refugio. Misiones: Iguazú, San Ignacio. Entre Ríos: La Paz. (MLP). Misiones: Posadas. Corrientes: Ita Ibaté, Paso de la Patria, San Cosme. Santa Fé: Garay (MACN).

## Discussion

The literature provided information on other species of Proconiini found in Argentina such as: *Acrogonia flaveoloides* Young, *Homalodisca ignorata* Melichar, *Molomea cincta* (Signoret), and *Phera centrolineata* (Signoret) (Gravena et al. 1998; Remes Lenicov et al. 1999; Dellapé and Paradell 2011).

The species *Diestostemma bituberculata* (Signoret), *Molomea vermiculata* (Signoret), *Molomea xanthocephala* (Germar), and *Stictoscarta sulcicollis* (Germar) were cited for Argentina by Young (1968) and Metcalf (1965), but none of them describe the province or locality where the specimens were collected.

The Proconiini, as other xylem feeding leafhoppers, are considered polyphagous and have evolved with many unusual adaptations, such as host switching, to maximize nutrient uptake (Mizell and Andersen 2001). New associations with host plants were found for 11 Argentinean sharpshooters (27.5%); the cited host plants belong to 24 families (Alliaceae, Apiaceae, Apocynaceae, Asteraceae, Bignoniaceae, Commelinaceae, Convolvulaceae, Fabaceae, Lamiaceae, Malvaceae, Meliaceae, Moraceae, Myrtaceae, Oleaceae, Oxalidaceae, Plantaginaceae, Polygonaceae, Poaceae, Rutaceae, Salicaceae, Sapindaceae, Solanaceae, Urticaceae, and Verbenaceae). Both known and new data of host pi ants-sharpshooter associations are summarized in Table 2.

The knowledge about natural enemies of Proconiini in Argentina is insufficient. Sharpshooter species are attacked by egg predators (Dermaptera), entomopathogenic fungus (Ascomycota) (Mariani et al. 1997; Toledo et al. 2006), and several egg parasitoids belonging Trichogrammatidae and Mymaridae families (Hymenoptera). In recent times, investigations conducted to survey the egg parasitoids of the Proconiini sharpshooters resulted in a greater and more comprehensive understanding of egg parasitoid wasps; the majority of the representatives of this guild belong to *Gonatocerus* Nees (Mymaridae), a wellknown genus showing a certain degree of specificity at level-tribe, because most of its species attacks Cicadellini and Proconiini sharpshooters (Triapitsyn et al. 2010). New records of parasitoids were found for 10 species (25%). Information of known natural enemies and new data are summarized in Table 3.

In Argentina, all the species of sharpshooters were found in two zones to north of latitude 40° S: one strip that connects the northeast with the mid-east of the country, and another from the northwestern to the mid-west (Figure 1). The most diverse genera (e.g., *Aulacizes* and *Oncometopia*) were found in both places. The eastern fringe includes the Paraná forest and was the most diverse; this is deeply linked to biogeographic systems of the Brazilian territory, which has the greatest diversity of Proconiini (Dellapé et al. 2011). All the studied sites where sharpshooters were found were grouped into the corresponding biogeographic provinces (sensu Morrone 2001, 2006) (Table 4).

**Figure 2.**
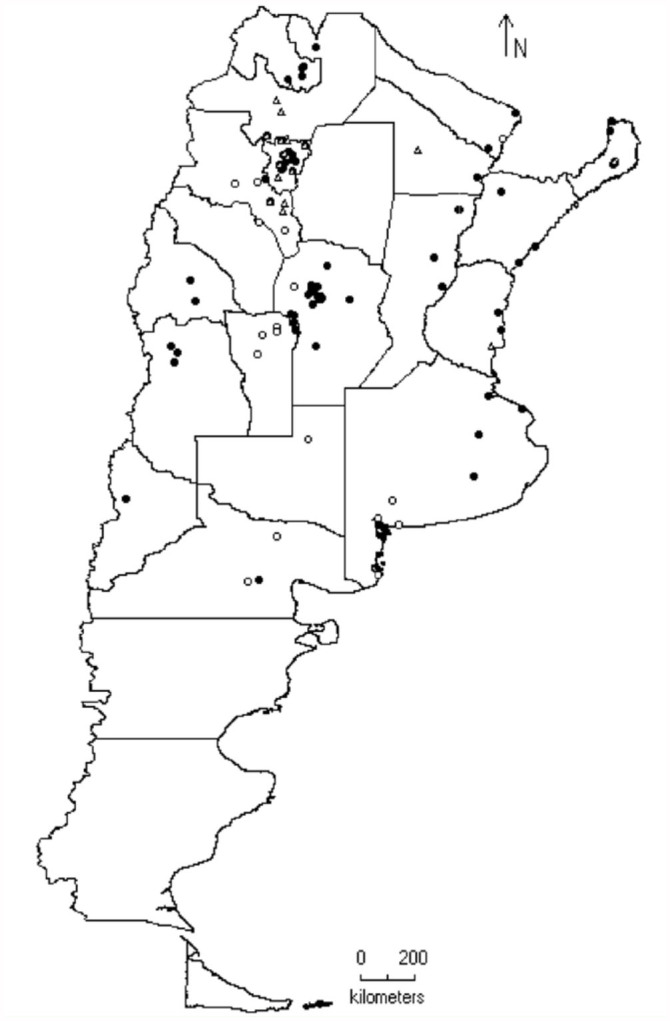
Distribution of the species of genus *Tapajosa* Melichar: *T. doeringi* (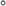), *T. rubromarginata* (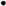) and *T. similis* (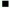). High quality figures are available online.

*Tapajosa* Melichar, the most widely distributed genus, was found in all the biogeographic provinces (except in the Puna); both *T. rubromarginata* and *T. doeringi* were the species with southernmost distributional range (Figure 2). *Tapajosa rubromarginata* was the most frequent and ubiquitous species, which was found in 70 localities of the Argentinean territory.

**Figure 3.**
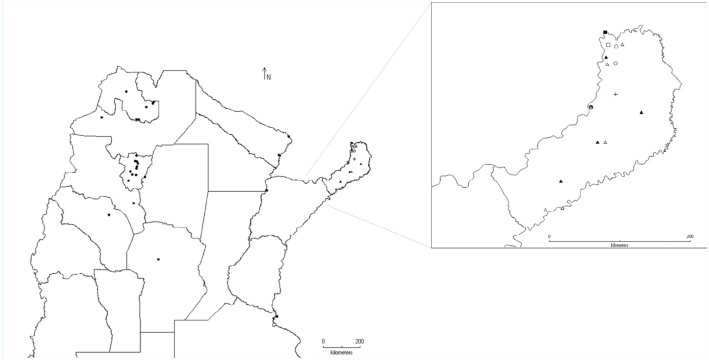
Distribution of the genera *Cicciana* Metcalf (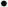), *Dechacona* Young (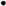), *Diestostemma* Amyot and Serville (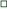), *Egidemia* China (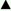), *Homalodisca* Stål (+), *Phera* Stål (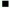), and *Teletusa* Distant (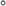). High quality figures are available online.

Six genera (*Cicciana* Metcalf, *Diestostemma* Amyot and Serville, *Egidemia* China, *Homalodisca* Stål, *Phera* Stål, and *Teletusa* Distant) were restricted to Paraná Forest—an evergreen forest with altitudes between 500 and 1800 m a.s.l., characterized by abundant trees over 30 m, Bambuceae, and arbustive ferns (Cabrera and Willink 1973) (Figure 3). The monotypic genus *Dechacona* Young was widely distributed in the northern part of the country, with a broad altitudinal range (from 60 to 4000 m a.s.l.) (Figure 3).

**Figure 4.**
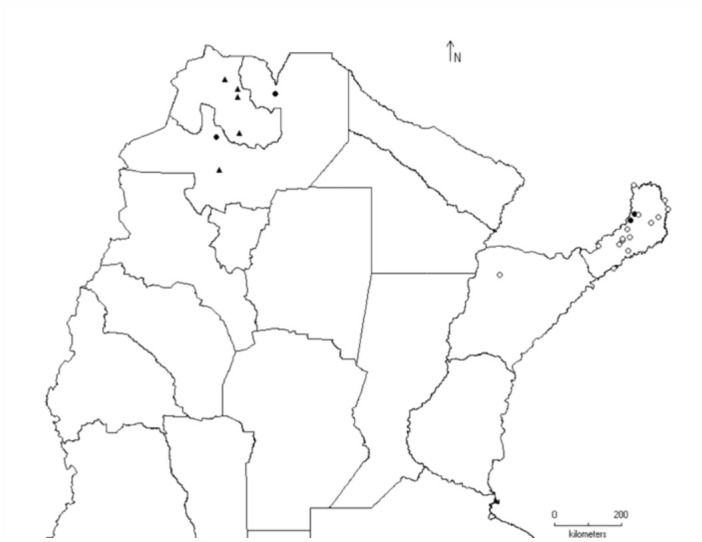
Distribution of the genera *Acrogonia* Stål (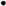), *Anacuerna* Young (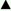), and *Aulacizes* Amyot and Serville (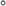). High quality figures are available online.

Three other genera were found in two biogeographic provinces: *Acrogonia* Stål (associated with jungle environments, both in Paraná and Yunga forest), *Anacuerna* Young (distributed in high elevations of Yunga and Puna), and *Aulacizes* Amyot and Serville (linked to forest environments and very humid localities of Chacoan subregion on the shore of the “Esteros de Iberá”) (Figure 4).

**Figure 5.**
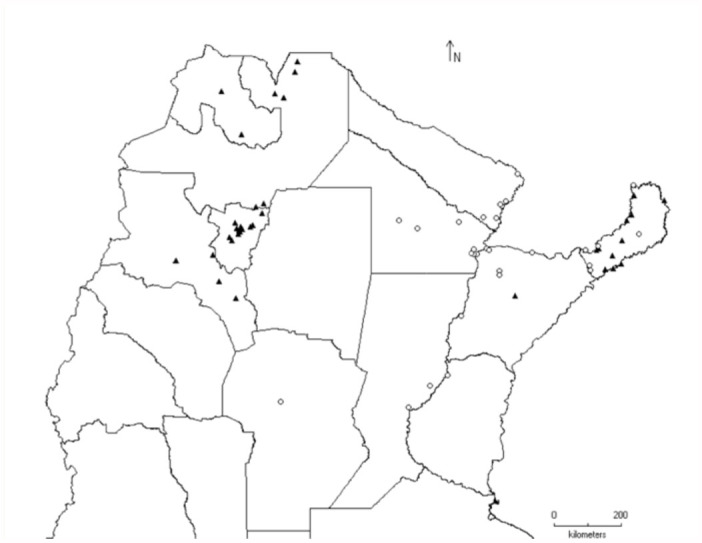
Distribution of the genera *Oncometopia* Stål (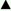) and *Tretogonia* Melichar (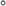). High quality figures are available online.

**Figure 6.**
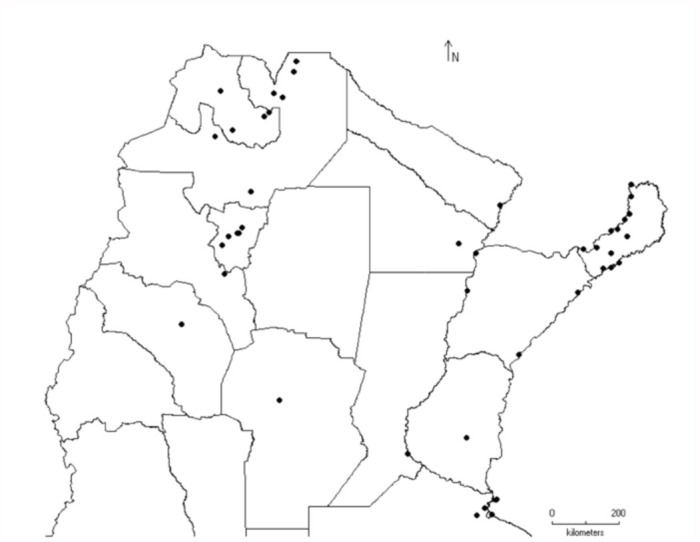
Distribution of the genus *Molomea* China (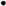). High quality figures are available online.

The genus *Tretogonia* Melichar (Figure 5) was found in sites of the Chaco province, with *T. notatifrons* being its most widely distributed species. *Oncometopia* Young is the genus with more species and was mostly linked to forest sites (Figure 5), but the species *O. facialis* and *O. tucumana* seemed to have more plasticity, occurring in four biogeographic provinces and a variable range of altitudes. Species of *Molomea* China were found in six different biogeographic provinces, with *M. consolida* having the widest range, as it was found to occur in 27 localities, from 60 to 2100 m a.s.l. (Figure 6).

Considering the 40 species of sharpshooters inhabiting the Argentinean territory, 19 of them (47.5%) were found only in the Paraná forest, and three species (7.5%) occurred only in the driest region of Chaco. The high elevation of Puna hosted only two species as well as Central Patagonia, where the specimens were collected in oasis located along river valleys.

The number of shared species between biogeographic provinces was low. The range of values of the Jaccard index varied between 0–1, representing complete dissimilarity between sampling for any taxon to a perfect match between sampling, respectively. The highest Jaccard index was obtained for adjacent provinces like “Prepuna-Yunga” (0.6), “Chaco-Pampa” (0.53), and “Prepuna-Monte” (0.5), while there were no shared species between “Puna-Pampa” and “PunaCentral Patagonia” (0), located very far from each other (Table 5).

This is the most comprehensive compilation of information related to species of sharpshooters in Argentina. The need for knowledge of interrelationships of insect pests and their environment has been emphasized by several authors in order to develop effective management tactics. In this context, the information given in this study could be useful for those involved in vector-control related programs.

**Table 1.**
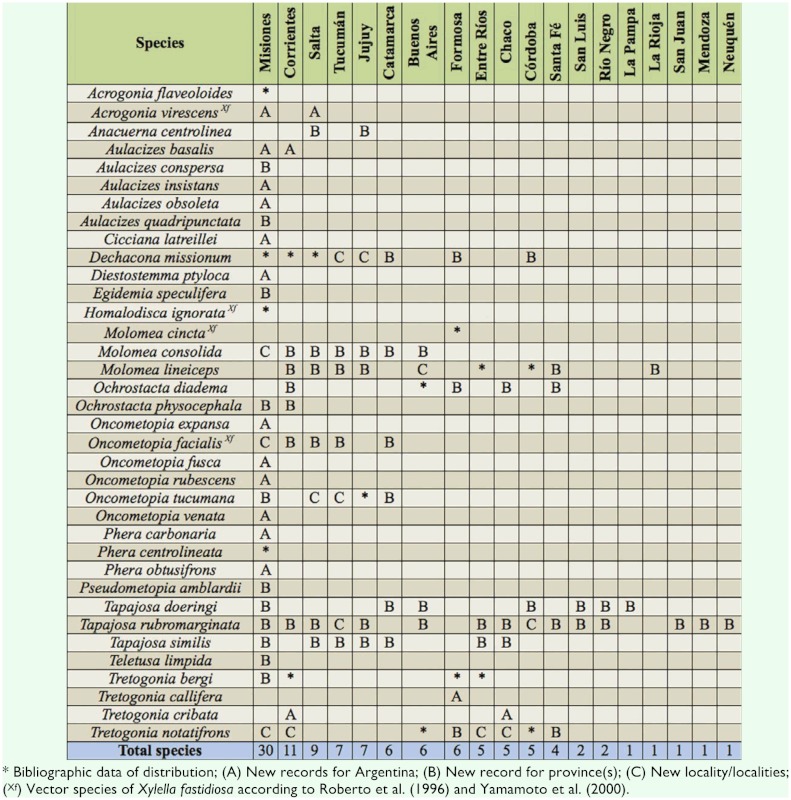
Geographic distribution of the Proconiini in Argentina by provinces, according to political divisions. The species *Diestostemma bituberculata, Molomea vermiculata, M. xanthocephala*, and *Stictoscarta sulcicollis* are not listed due to the lack of information about the collection site.

**Table 2.**
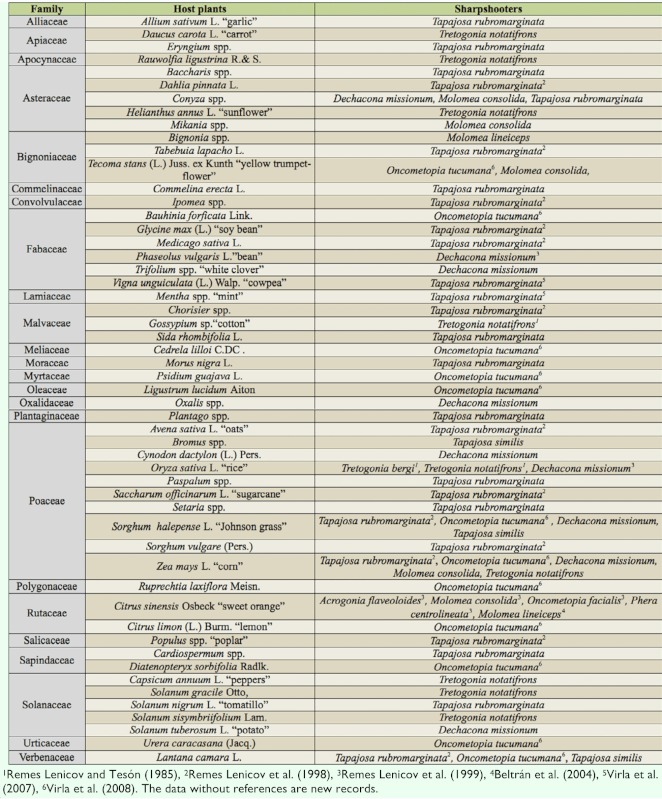
Host plants records of the sharpshooters occurring in Argentina.

**Table 3.**
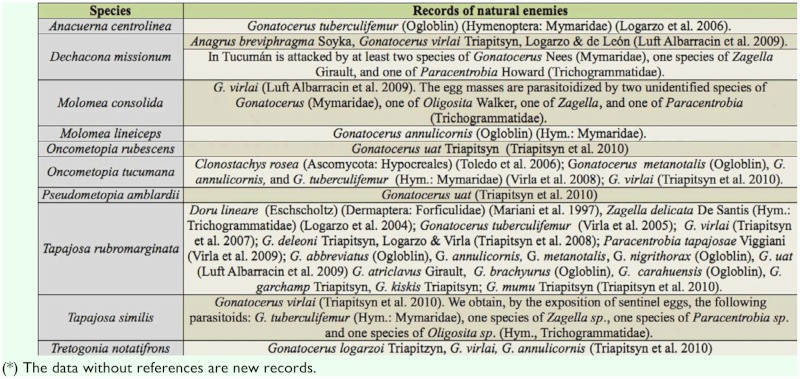
Summarized records of natural enemies of the Argentinean Proconiini sharpshooters (*).

**Table 4.**
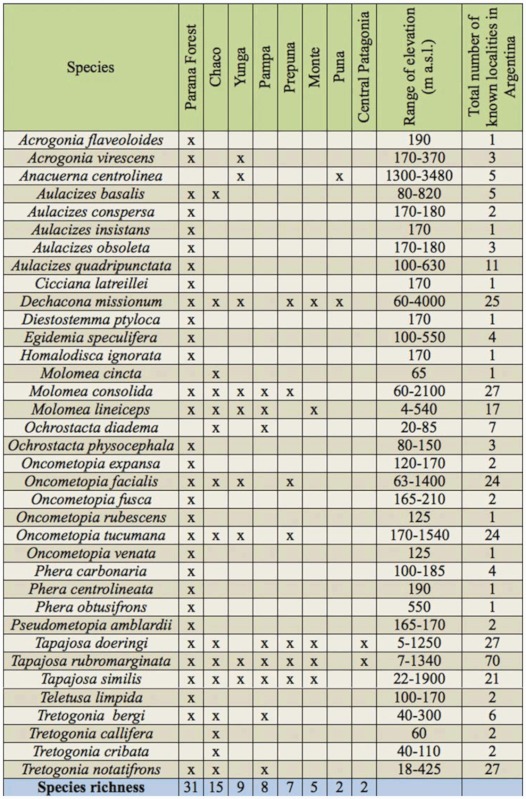
Distribution of the Argentinean Proconiini sharpshooters into the biogeographic provinces (according to Morrone 2001, 2006). The range of elevation of the localities in which each species occurs is given.

**Table 5.**
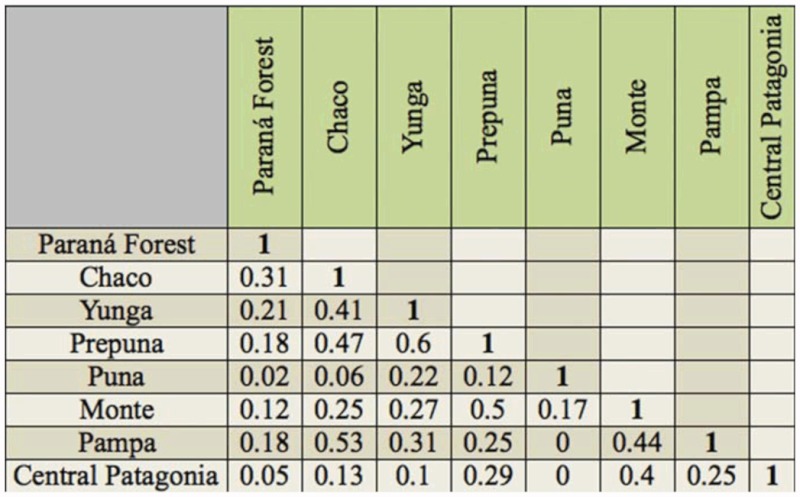
Matrix of Jaccard Similarity Coefficient between Argentinean biogeographic provinces hosting Proconiini sharpshooter species.

## Editor's note

Paper copies of this article will be deposited in the following libraries. Universitaetsbibliothek Johann Christian Senckenberg, Frankfurt Germany; National Museum of Natural History, Paris, France; Field Museum of Natural History, Chicago, Illinois, U.S.A.; University of Wisconsin, Madison, Wisconsin, U.S.A.; University of Arizona, Tucson, Arizona, U.S.A.; Smithsonian Institution Libraries, Washington D.C, U.S.A.; The Linnean Society, London, England. The date of publication is given in ‘About the Journal’ on the JIS website.
